# Action after Adverse Events in Healthcare: An Integrative Literature Review

**DOI:** 10.3390/ijerph17134717

**Published:** 2020-06-30

**Authors:** Mari Liukka, Alison Steven, M Flores Vizcaya Moreno, Arja M Sara-aho, Jayden Khakurel, Pauline Pearson, Hannele Turunen, Susanna Tella

**Affiliations:** 1 Department of Nursing Science/Faculty of Health Sciences, University of Eastern Finland, 70211 Kuopio, Finland; hannele.turunen@uef.fi (H.T.); susanna.tella@lab.fi (S.T.); 2 South Karelia Social and Health Care District, 53130 Lappeenranta, Finland; 3 Department of Nursing, Midwifery and Health, Northumbria University, Newcastle upon Tyne NE7 7XA, UK; alison.steven@northumbria.ac.uk (A.S.); pauline.pearson@northumbria.ac.uk (P.P.); 4 Faculty of Health Sciences, University of Alicante, 03690 Alicante, Spain; flores.vizcaya@ua.es; 5 Faculty of Health Care & Social Services, LAB University of Applied Sciences, 53850 Lappeenranta, Finland; arja.sara-aho@lab.fi; 6 Research Center for Child Psychiatry, University of Turku, 20500 Turku, Finland; Jayden.khakurel@utu.fi; 7 Clinical Development, Education and Research Unit of Nursing (CDERUN), Kuopio University Hospital, 70210 Kuopio, Finland

**Keywords:** patient safety, adverse events, first victims, second victims, third victims, management

## Abstract

Adverse events are common in healthcare. Three types of victims of patient-related adverse events can be identified. The first type includes patients and their families, the second type includes healthcare professionals involved in an adverse event and the third type includes healthcare organisations in which an adverse event occurs. The purpose of this integrative review is to synthesise knowledge, theory and evidence regarding action after adverse events, based on literature published in the last ten years (2009–2018). In the studies critically evaluated (*n* = 25), key themes emerged relating to the first, second and third victim elements. The first victim elements comprise attention to revealing an adverse event, communication after an event, first victim support and complete apology. The second victim elements include second victim support types and services, coping strategies, professional changes after adverse events and learning about adverse event phenomena. The third victim elements consist of organisational action after adverse events, strategy, infrastructure and training and open communication about adverse events. There is a lack of comprehensive models for action after adverse events. This requires understanding of the phenomenon along with ambition to manage adverse events as a whole. When an adverse event is identified and a concern expressed, systematic damage preventing and ameliorating actions should be immediately launched. System-wide development is needed.

## 1. Introduction

Adverse events (AEs) are inevitable in nursing and healthcare [1,2]. Even where best professional care exists, most treatments or investigations have the potential to cause harm [3]. Although the culture and system of a healthcare organisation (HCO) may be well developed, AEs will happen because of human factors and HCOs being complex adaptive systems, always changing and evolving. Thus, comprehensive preparation is important both to minimise harm to victims and to maintain the functionality of HCOs. In organisations with positive patient safety cultures professionals can speak openly about issues and events without fear of blame or punishment. Managers promote safety and reporting of AEs is supported and organisational learning occurs [1]. 

An AE is defined as an unintended or unexpected incident which causes harm to a patient and may lead to temporary or permanent disability [1,4]. Approximately every tenth patient in hospital suffers such events [5]. A quarter of these events in Europe are healthcare-associated infections; other AE types include medication errors, surgical errors, diagnostic errors, medical device failures or failure to act on test results [6]. Nurses and healthcare professionals often witness or are involved in AEs [2,7,8]. In healthcare, AEs can, at worst, cause catastrophic consequences [1]. It is clear that taking action after an AE has occurred is as important as prevention. About half of physicians say that involvement in AE increases stress in their work [9]. Many of the second victims seek support from family, colleagues or supervisor [10]. About 10% agree that organisations support them in coping with AEs [9].

Three kinds of victims of AEs can be identified. The “first victims” are conceptualised as patients and their families. Patients can suffer from an AE in two ways: first from direct harm caused and then from the way the event is handled [1]. The “second victims”, a concept originally introduced by Wu [11], are healthcare providers, including physicians, nurses, allied clinicians, support personnel, students and volunteers [12], who have been involved in a patient related AE and subsequently experience emotional or physical distress, thus becoming a victim themselves [13,14]. The phenomenon is quite common: the prevalence of second victim suffering is anticipated to be approximately 30%, varying from 10.4% to 43.3% [15]. Ninety per cent of healthcare professionals reported suffering at least one physical or psychosocial “second victim” symptom [16]. The “third victims” are healthcare organisations in which the AE occurs [17]. The impact on third victims can also be considerable, as AEs may create an organisational crisis leading to long-term business difficulties [18].

The effects of an AE on first, second and third victims include health-related, functional and economic consequences. These are interrelated and can cause significant costs. Both the first and second victims may suffer emotional and psychological, physical, financial and livelihood consequences [19]. In addition, second victims can face professional consequences, including concerns regarding the performance of their work [12,15,20,21,22]. Healthcare professionals may also experience difficulties working in an environment where AEs have occurred [23,24]. Consequences for third victims relate to effectiveness [12,19,20], reputation [19,25], legal [20] and economic issues [19]. Hence, these phenomena are crucial aspects to consider after an AE.

Managing the aftermath of AEs well can be assumed to have positive consequences for first and second victims’ health, behaviour and economic well-being. Considering HCOs as third victims, but also as responsible for the first and second victims, it is clear that where possible systematic prevention of first and second victim consequences, and appropriate care after an AE is crucial. Constructive actions after an event can have a positive impact on the safety culture, effectiveness of services and financial situation of the HCOs. In the US, the estimated cost of medical error in 2008 was USD 1 trillion, but patient safety improvements are estimated to have saved USD 28 billion [26]. Strategies to reduce the rate of AEs in the European Union alone could prevent more than 750,000 harm-inflicting medical errors per year. That means over 3.2 million fewer days of hospitalisation, 260,000 fewer incidents of permanent disability and 95,000 fewer deaths per year [27]. The economic consequences of AEs, and of how the events are handled, are therefore not limited to healthcare. For nations, increased absence from work, staff leaving the professions and deaths are examples of extreme consequences of AEs. Actions after AEs can be assumed to have serious short- and long-term, direct and indirect impact on individuals, the economy and society.

The purpose of this integrative review is to synthesise existing knowledge on actions following AEs in HCOs such as hospitals and primary care units. The aim is to identify the underlying elements required for damage preventing and ameliorating actions following AEs in order to provide direction for development and future investigation. The research question is: What are the key elements of action immediately after AEs in HCOs? 

## 2. Materials and Methods 

### 2.1. Design of the Study

An integrative review approach was used following Whittemore and Knafl’s five stages: (1) the problem was identified; (2) the relevant literature published between 2009 and 2018 was sought; (3) the screened data were evaluated using a 10-item tool; (4) the eligible data were analysed using inductive content analysis; and (5) the findings are presented in tables [28]. In addition, the checklist of the Preferred Reporting Items Systematic Reviews and Meta-analysis (PRISMA) Statement (2009) was used to guide the review [29].

### 2.2. Search Strategy

The databases Scopus, CINAHL, Cochrane and PubMed were searched for relevant articles. Boolean search methods were used to retrieve articles related to action after adverse events in healthcare such follows: “adverse event” AND “disclosure” OR “aftermath”, “adverse event” AND “professional’ support”, “healthcare” AND “second victim”, “healthcare” AND “after error”.

**Figure 1:**
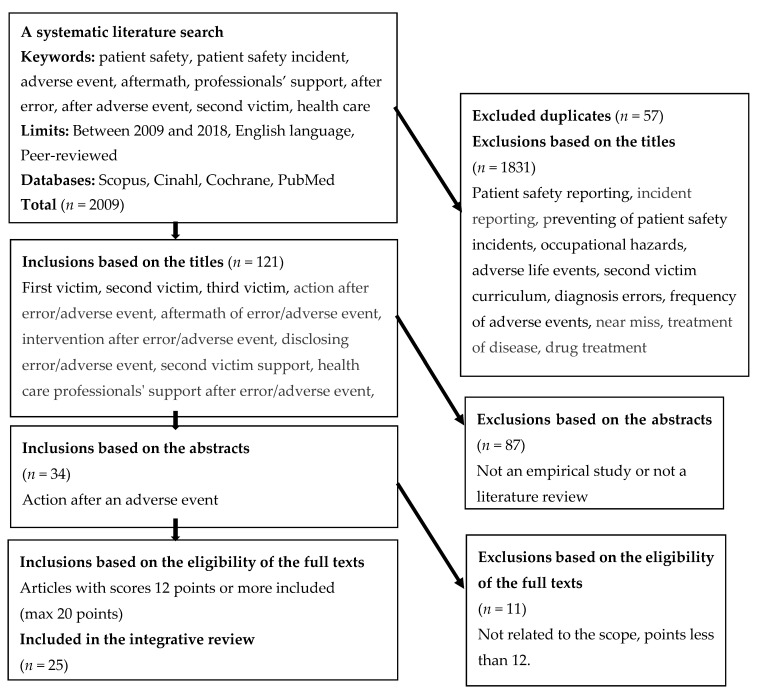
Systematic literature search process regarding action after adverse events.

The search, for example, from Scopus included search terms “adverse event” AND “aftermath” OR “disclosure” with limits “in article, title, keywords”, “published 2009 to 2018”, “article or review”, “English language” and “in journals”. Articles were included if they reported on action after AE. Articles focusing on, for example, adverse drug reactions or AE reporting were excluded. Articles about AE reports were excluded when they were only about frequency of reports, or near misses and did not present the whole process from AE to disclosure. Search methods, inclusion and exclusion criteria and search outcomes are presented in Figure 1. Twenty-five research or review papers were found for inclusion in the data evaluation process. 

### 2.3. Review and Quality Assessment Process

The search process was realised independently by the authors (ML and ST). Online discussions were held with other authors to share results and make decisions on next steps of the process.

The “quality” of papers was evaluated using a tool developed from an amalgamation of previous work [30,31,32,33] which was refined via international research group discussions. The evaluation areas included: (1) background; (2) aim and research questions; (3) sample; (4) data collection; (5) data analysis; (6) results; (7) ethical issues; (8) reliability; and (9) usefulness of the results. After discussing relevant evaluation areas for a comprehensive quality assessment, the research group added a further area: (10) strengths and limitations. Each evaluation area was scored from 0 to 2 points using the following criteria: (0) does not meet the aim or lacks data; (1) inaccurate or superficial; and (2) relevant and presented systematically. With 10 evaluation areas and a maximum of 2 points for each area, the range of the scores for a study varied from 0 to 20 points. Anything below 12 points was excluded due to low quality.

The articles retrieved were distributed evenly, and two researchers independently scored each paper using the tool. Total scores for each paper were compared and the content, importance, face validity and quality of each paper discussed. Where differences of three points or more were present, each sub-element score was discussed, and a third research team member acted as a moderator to arrive at a consensus. Cohens’ Kappa was calculated to test interrater reliability (κ = 0.83).

### 2.4. Data Analysis

The results of the studies retrieved were analysed using inductive content analysis [34]. First, the studies were read several times and listed in a table to gain an understanding of the whole and the characteristics of the actions taken after an AE. The data reduction phase included extraction of the data into a manageable framework. The aims of the study, research methods, findings, scores and scope of the action after AEs were presented. Then, the data were open coded, abstracted and categorised using content-characteristic words. Sub-categories were developed and discussed in the international research group. Sub-categories were further grouped into categories describing management of action after AEs. Care was taken not to double count data from individual studies duplicated in literature reviews.

## 3. Results

### 3.1. Characteristic for the Studies

The papers retrieved (*n* = 25) were published between 2009 and 2018 (Table 1). The largest numbers of papers were published in 2015 (*n* = 5) and 2018 (*n* = 5) and were from the USA (*n* = 12). Various methodologies were present: quantitative (*n* = 10), qualitative (*n* = 8), multiple methods (*n* = 2) and literature reviews (*n* = 5). The quality scores of the papers varied from 12 to 20 points, with a mean of 15.9 and standard deviation 2.1. The majority (*n* = 21) of papers were about second victim phenomenon and less attention was given to first (*n* = 6) and third victim phenomena (*n* = 4). One paper encompassed both first and second victims, three included both second and third “victims” and one paper covered all three “victims”.

### 3.2. Key Elements of Responses and Action after AEs Bulleted Lists Look Like This

Actions following AEs were comprised of three themes, namely first victims, second victims and third victims, with empathic and ethical communication, support services, complete apology and training and learning as cross-cutting elements.

The theme of action for first victims was comprised of four elements: attention in revealing an AE, communication after AEs, first victim support and complete apology (Table 2). Patients and families [19] and healthcare providers [35,36] alike were often afraid of speaking up. Empathic, ethical and open communication played an important role overall; the quality of the communication seemed to either empower or disempower patients and their families [19,37,38,39]. In many cases, patients are not informed about AEs [40]. Support for first victims was addressed primarily as a lack or neglect of emotional support [36,39] and compensation support [35]. Apologising was an important element after experiencing an AE [19,34,37,38]. First victims perceived the apology as an integrative process, where the style and the presenter of the apology, whether healthcare provider or organisation, played an important role. Expressing empathy, giving honest information about the AE, taking responsibility and learning from the event were crucial to the apology process.

The action for second victims theme consisted of the following elements: second victim support types, coping strategies, support protocols, changes after AEs and learning about AE phenomena (Table 2). Support types consisted of informal [12,15,41,42,43,44,45], formal [15,23,25,40,41,46,47] and emotional [22,42,44,45,46] support for second victims. Healthcare providers have indicated informal peer support as important [20,41,42,49,50], but sensitive. The support can be destroyed, for example, by blaming, gossiping and silence [46]; thus, it is important to pay special attention to non-blaming, open and supportive communication. Formal support was not a certainty and was not offered in all cases [12,25,42,46,47]. The importance of emotional second victim support was clear and could be provided for all those involved, for individuals or groups [43,49,50]. Second victim coping strategies related to the individuality of strategies [12,49], emotional support [41,47,49,51] and problem solving [47,49]. 

The second victim support services comprised availability [11,24,25,41,44], counselling support [36,41,44], time away support [41,44,45] and open disclosure support [37,43,44]. Changes that second victims make after an AE can include defensive and constructive changes [50]. It was also found that learning about AEs [47], the second victim phenomenon and learning to communicate about AEs are important for staff members [12,44,48].

**Figure 2:**
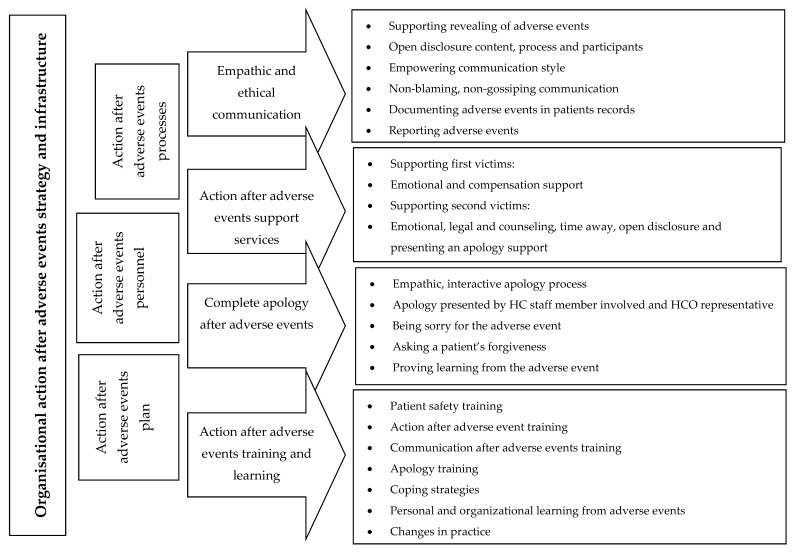
“Action after adverse events” in healthcare organisations.

The action for the third victims theme consisted of organisational strategy and infrastructure [20,46,49], which was divided into action after adverse events plan [12,25,52], personnel [36,37,42,46,52] and processes [20,36,52] subthemes (Figure 2). The key elements of the subthemes were:

- emphasising open, empathic communication (for example, open disclosure) and each staff member’s responsibility for their empowering communication style [25,37,42];
- action after AE support services for first and second victims (for example, emotional support) [42,44,47,49]; and
- action after AE training and learning for managers and staff members [15,19,52].

## 4. Discussion

The results of this integrative literature review demonstrate how complex and multi-layered the phenomenon “action after AE” is and how this topic has gained attention in international research and healthcare development work. Previous studies have concentrated more on a single perspective regarding actions after AEs, while, in this integrative review, a more holistic view is presented. Key themes emerged relating to victims of AEs: first, second and third victim elements, with empathetic, effective communication, support services, complete apology and training and learning, as cross-cutting elements.

The first victim theme comprised attention to revealing an AE, communication after an event, first victim support and complete apology. The second victim theme included second victim support types, coping strategies, support services, changes after AEs and learning about AE phenomena. The third victim theme consisted of organisational action after AEs, strategy, infrastructure and training and open communication about AEs. These three themes interweave tightly together, and we approach the themes from a healthcare organisation’s perspective to outline the needs of first and second victims and how HCOs could respond to these. In this integrative review, second victim support programs were under development work. For example, Scott et al. designed “A Framework of Caring: The Scott Three-Tiered Interventional Model of Support”, which features: (Tier 1) unit level support; (Tier 2) trained peer supporters and patient safety and risk management resources; and (Tier 3) an expedited referral network with specialist support [12]. Indeed, a similar kind of support program could also benefit first victims.

Second victim support programs can be assumed to support first victims as well through better preparation of nurses and healthcare providers. However, it could be argued that more comprehensive first victim support programs are also needed. Attention to revealing an AE, open and emphatic communication and complete, authentic apology to, and support of, first victims were essential after AEs. For example, the apology policy of the HCOs seemed to be fragmented and often defensive. First victims highlighted the importance of an empathic, interactive process, where a sincere apology is expressed not just by an individual healthcare provider, but responsibility on the part of the HCO is accepted as well [53,54]. First victims implied that in some situations they might forgive, but it was unclear if forgiveness was asked for [35]. Here, an interactive support program could be beneficial for all victims, including nursing and healthcare students. For instance, first victims wanted the apology to include information about how the HCO would learn from the AE and make changes [19,35]. First victims had often lost trust in HCOs [19]. Open discussion about what went wrong, and why, can be the first step to understanding and forgiveness [55]. One reason for a loss of trust may be a lack of transparency after AE [56]. First victims should be convinced that everything possible is being done to avoid a similar situation in the future. If the apology included a convince of systematic, organisational level learning from the AE, the professionals involved may feel supported when discussing AEs with patients, peers and managers [57]. From the literature reviewed changes appear needed at the individual, team, unit and organisational levels. The results suggested a need for holistic approaches to managing AEs.

Safe, systematic and clear “action plan after AEs” required an understanding of each stakeholder’s needs. AEs consist of complex systems of problems which often interact; thus, it is important to deal with the phenomenon as a whole. Indeed, even those not directly involved may have impact on the consequences of AEs. The strategy and infrastructure of HCOs are crucial to managing action after AEs as part of healthcare delivery. An “action after AE” strategy needs to include a comprehensive plan which attends to the interlinked complexity which often exists. Well-thought-through communication is required from everyone in HCOs: colleagues, managers and second victims as well. AEs are very sensitive events that can have long-term consequences [12,15,19,20,24]. Thus, communication is fundamental to occupational and patient safety.

Organisational “action after AEs” infrastructure needed to have appointed personnel, clear support and learning infrastructure and clear processes. It was also important that the process and content of open disclosure are included in the management of the events. Emphatic, support and respect by colleagues is needed after AE so that healthcare professionals still feel competent to do their job [20]. With these actions, HCOs may be able to ameliorate the severe consequences for all victims, such as effectiveness of HCOs [12,19,20], economic issues [19] and reputation [19,25]. Nurses and healthcare professionals suffer when involved in AEs, may fear reporting events [48,58,59,60] and experience difficulties working in an environment where AEs have happened [23]. Being comprehensively prepared is important [58] both to minimise harm to all victims and for the functionality of healthcare systems. 

Mira et al. found that many patients are not informed at all about AE. This may be because HCPs are afraid for their professional future, or because they do not have competence to honestly tell a patient what has happened [38,40,51]. A shortage of skill and resource lack of competence seems to be one barrier to developing organisational support programs after AE [50]. It is important not to forget the first victims outside this support. It is also good to recognise that first victims have much information about AEs to provide for organisational learning [38,39]. Crucial for this is that action after AE education is included in professional and continuing healthcare programme [33].

The strengths of this study include an international researcher group involved with strong patient safety research, management and education experience. For example, the data evaluation was conducted in two groups. The quality of the research papers was evaluated with an instrument used in an integrative review. Agreement among authors was measured by Cohen’s kappa (κ = 0.411), which can be interpreted as moderate [60]. Limitations include the method itself. Only peer reviewed research papers were used in this review. National or international guidelines and protocols about disclosing adverse events were omitted. The search strategy may have affected the number of different victim phenomena found vary. Combining different methodologies such as qualitative, quantitative and literature reviews can be difficult due to diverse ontological and epistemological underpinnings, which some may view as causing bias [28]. Team discussions regarding key features of the papers were utilised to assist in clarifying the quality of the studies and the main emergent points from each paper. Close attention was also given to the avoidance of double counting in order to avoid “skewing” the findings. The PRISMA statement was used to guide the writing of the review [29].

## 5. Conclusions

It is inevitable that AEs will occur in healthcare organisations, impacting on individual, team, unit, organisation and national levels. When an AE is identified and a concern expressed, immediate and comprehensive action should be taken. This requires trying to understand the whole phenomenon in its complexity, an ambition to manage AEs and a “just restorative” culture [61] that enables it. System-wide developments are needed regarding action after AEs, along with the implementation of evidence-based organisational infrastructures and strategies which could ameliorate the suffering of patients, their families and healthcare providers, as well as help healthcare organisations (and ultimately nations) to use resources effectively. For this developing, more research about patients’ and their families’ needs as well as organisations’ needs is required. Tight collaboration is needed between policy-makers, nursing and healthcare managers and educators in order to develop such systems and the necessary culture [62]. Only then will all victims receive appropriate support after AEs. We also suggest that future education, research, policy and practice developments should incorporate a move to a more balanced approach incorporating both Safety 1 (learning from failure) and Safety 2 (learning from how things typically go right) perspectives [61]. At the national level, social and healthcare ministries are responsible for planning, guidance and implementation of health and social policy to safeguard people’s ability to work and function. International collaboration between governments is needed to standardise studies concerning first, second and third victim phenomenon. Governments should build a network of researchers and healthcare managers for developing the study protocols and shared understanding of developing first, second and third victim support system in healthcare organisations. Such a move may assist in the development of “restorative just cultures” in HCOs and more holistic approaches to actions after AEs for the benefit of all “victims”.

## Figures and Tables

**Table 1:** Studies investigating action after adverse event.

Author(s) (Year), Country	Purpose and Aims of the Study	Research Methods/ Instrument/Sample (*n* = 25)	Findings	Evaluation Scores/Scope
Scott et al. (2010), USA [12]	To describe a deployment of an institutional rapid response system (RRS) for second victims	Interview and 10 item web-based survey Interviews with 31 healthcare professionals Survey (*n* = 898), medical students, physicians and professional nurses	Six distinct recovery stages were delineated. Almost 40% of the respondents had previously heard the term *second victim*; 30% have had personal problems within the past 12 months, such as anxiety, depression or concerns about their ability to perform their jobs. Thirty-five per cent of respondents reported receiving support from colleagues and peers when it was offered and 29% received support from supervisory personnel. Eight themes from the narratives to describe general support infrastructure characteristics to aid second victim recovery were identified.	12.5 Second victim
Seys et al. (2013a), USA [15]	To identify supportive interventional strategies for second victims	Literature review 21 research articles and 10 non-research articles Inclusion criteria and search strategy described PRISMA method was used for reporting	Numerous supportive actions for second victims described in the literature. Strategies included support organised at the individual, organisational, national or international levels. Second victim support is needed to care for healthcare workers and to improve quality of care. Support can be provided at the individual and organisational levels. Programs need to include support immediately post adverse event as well as on a middle- and long-term basis	14 Second victim
McVeety et al. (2014), Canada [19]	To analyse and synthesise best evidence on the perspectives of patients and family members who encountered adverse events	Review, 14 studies that used qualitative methodologies included Inclusion criterions and search strategy described, Joanna Briggs Institute Qualitative Appraisal and Review Instrument (JBI-QARI) and Appraisal Checklist for Interpretive and Critical Research	Nine themes were identified relating to patient and family perceptions and experiences of an adverse event: communication, the disclosure process, apology, consequences and impact, fear of reprisal and/or interference with care, learned helplessness, measures of safeguarding, self-discovery and awareness of errors, and violations of trust.	16 First victim
Ullström et al. (2014), Sweden [20]	To investigate how healthcare professionals are affected by their involvement in adverse events, with emphasis on the organisational support they need and how well the organisation meets those needs.	Semi-structured interview guide with 30 questions. Qualitative content analysis and systematic classification was used Healthcare professionals (*n* = 21)	Impact on the healthcare professional was related to the organisation’s response to the event.	15 Second and third victim
Kable et al. (2018) Australia [22]	To understand the effects of adverse events on nurses in acute health-care settings.	A qualitative, descriptive study design; 10 nurses, semi-structural interview.	Nurses need organisational responses to adverse events, including collegial support and provision of information after adverse event occur.	17 Second victim.
Rodriquez and Scott. (2018) USA [24]	To examine experiences of healthcare professionals who changed paths after an adverse event.	Web-based survey with total of 105 individual responded; 77 (73,3%) were eligible to complete the survey.	Healthcare professionals reported a pattern of inadequate social support after adverse event. More transparency and support to help professionals recover is needed.	14 Second victim
Mira et al. (2015a), Spain [25]	To identify and analyse organisation-level strategies adopted in both primary care and hospitals in Spain To address the impact of serious AE on second and third victims	A cross-sectional survey study. The questionnaire explored five intervention areas: safety culture; health organisation crisis management plans for serious AE; measures to ensure transparency in communication with patients (and relatives) who experience an AE; care and support for second victims and actions to protect the reputation of the health organisation (the third victim). Developed by consensus among the research team on the basis of reviews Managers of hospital and primary care centres (*n* = 197), patient safety coordinators in hospitals or primary care (*n* = 209)	Deficient provision of support for second victims was acknowledged by 71% and 61% of the participants from hospitals and primary care, respectively; these respondents reported that there was no support protocol for second victims in place in their organisations. Regarding third victim initiatives, 35% of hospital and 43% of primary care professionals indicated that no crisis management plan for serious AE existed in their organisation, and, in the case of primary care, there was no crisis committee in 34% of cases. The degree of implementation of second and third victim support interventions was perceived to be greater in hospitals (mean 14.1, SD 3.5) than in primary care (mean 11.8, SD 3.1) (*p* < 0.001)	17.5 Second and third victim
Gu and Itoh (2012), China [35]	To explore Chinese patients’ views on physician disclosure actions after an adverse event and their acceptance of different types of apologies from the physician who caused the event.	Questionnaire with seven sections concerning responding views of issue related to medical errors and patient safety Inpatients and families (*n* = 934)	A large difference identified in the level of patient acceptance between a physician’s “full” or “partial” apology. It is suggested that Chinese hospitals should adopt an “open” policy, which should include a “sincere” apology to the patient who experienced a medical error in order to maintain mutual trust between the staff and patients.	17 First victim
Mira et al. (2015b), Spain [36]	To assess the effect of adverse events that occur in primary care and hospital settings on health professionals in personal and professional terms	A cross-sectional study Online survey, randomly selected sample; 1087 health professionals completed the questionnaires (610 from primary care and 477 from hospitals)	In total, 430 health professionals had informed a patient of an error. Error reporting to patients was carried out by those with the strongest safety culture, under 50 years of age and primary care staff. Primary care (*n* = 318) and hospital (*n* = 346) health professionals reported having gone through the second-victim experience. The emotional responses were: feelings of guilt, anxiety, re-living the event, tiredness, insomnia and persistent feelings of insecurity. In doctors, the most common responses were feelings of guilt and re-living the event, while nurses showed greater solidarity in terms of supporting the second victim in both PC and hospital settings.	18 Second victim
Sorensen et al. (e-pub 2009), Australia [37]	To understand patients’ and health professionals’ experience of Open Disclosure and how practice can inform policy	Semi-structured open-ended interview. Grounded theory was used to analyse the data Nurses, managers, policy coordinators, patients and family members (*n* = 154)	Five major elements influenced patients’ and professionals’ experiences of openly disclosing adverse events namely: initiating the disclosure, apologising for the adverse event, taking the patient’s perspective, communicating the adverse event and being culturally aware.	15.5 First and second victim
Koller and Espin (2018) Canada [38]	To capture perspectives on paediatric disclosure and identify gaps in knowledge for best practices and policy uptake.	Focus group interview with semi-structured questions; 5 parents, 14 children and adolescents and 27 healthcare providers.	Patients and families need full disclosure and right to know about errors. Health-care professionals need more clarity in policies. Most agreed that a case-by-case approach was necessary for supporting variations in how medical errors are disclosed.	19 First victim
Hågensen et al. (2018) Norway [39]	To present patients’ perspectives of disclosure of and healthcare organisations’ response to adverse events.	Qualitative study; 15 in-depth interviews.	Three main topics regarding patients’ experiences of adverse events are: (1) ignored concerns or signs of complications; (2) lack of responsibility and error correction; and (3) lack of support, loyalty and learning opportunities.	20 First victim
Mira et al. (2017), Spain [40]	To summarise the knowledge about the aftermath of adverse events and to develop a recommendation set to reduce their negative impact in contexts where there is no previous experience and apology laws are not present.	Three information sources were used; review studies (*n* = 14 publications), institutional websites (16 websites were reviewed) and experts’ opinions and experience on patient safety (four focus group sessions with 27 participants).	Recommendations focused on eight areas: (1) *Safety and organisational policies*; (2) *Patient care*; (3) *Proactive approach to preventing reoccurrence*; (4) *Supporting the clinician and healthcare team*; (5) *Activation of resources to provide an appropriate response*; (6) *Informing patients and/or family members*; (7) *Incident analysis*; and (8) *Protecting the reputation of health professionals and of the organisation*.	19 First, Second and third victim
Treiber et al. (2018) USA [41]	To discuss the second victim syndrome and its impacts on nurses.	Online survey with multiple-choice and open-ended items were sent to 842 resent nursing graduates 168 responses were received.	Fifty-six per cent reported making at least one medication error. After making a medical error nurses had emotional responses, such as fear and disappointment. Nurses described often been supported by peers, nursing manager and preceptors.	12 Second victim
Burlison et al. (2017), USA [42]	To present the development and psychometric evaluation of the Second Victim Experience and Support Tool (SVEST), a survey instrument that can assist healthcare organisations to implement and track the performance of second victim support resources	Quantitative study Second Victim Experience and Support Tool (SVEST) questionnaire development, 5-point Likert scale Nurses, physicians, pharmacists and medical technicians in specialised paediatric hospital (*n* = 305)	The SVEST (The Second Victim Experience and Support Tool) can be used by healthcare organisations to evaluate second victim experiences of the quality of existing support resources. Means: Psychological distress 2.6, physical distress 2.3, colleague support 2.2, supervisor support 2.8, institutional support 2.3, non-work-related support 2.4, professional efficacy 2.5, turnover intentions 2.1, absenteeism 1.8 The most desired second victim option: A discussion with a respected peer 81% The second most desired option: A discussion with the manager 74%	19.5 Second victim
Edrees et al. (2011), USA [43]	To emphasise the importance of support structures for second victims in the handling of patient adverse events and in building a culture of safety within hospitals.	A cross-sectional survey using a two-part Second Victim Questionnaire Nurses, nursing or other managers, physicians, pharmacists, therapists, clinical support, technologists (*n* = 140 in part one and *n* = 95 in part two)	There is *a need for second victim support strategy in healthcare organisations*. Informal emotional support and peer support are among the most requested and most useful strategies. Other desired support: Prompt debriefing, crisis intervention stress management (75%), an opportunity to discuss ethical concerns related to an event or process (46%), a safe opportunity to contribute to the prevention of similar events in the future (45%)	13.5 Second victim
Ferrús et al. (2016), Spain [44]	To identify what occurs among healthcare providers after an adverse event and what colleagues could do to help them	A qualitative study applying consensus search techniques Focus group and metaplan Physicians (*n* = 15), nurses (*n* = 12)	Consensus about second victims requiring support from their colleagues and managers; many times, second victims perceive rejection. They experience fear, repetitive thoughts and loneliness. Formal information channels favour implementation of improvements. HCPs perceived that information on measures for preventing another adverse event is inaccessible. Managers reported that a change in behaviour is necessary to improve patient safety culture. Common informal channels included cafeterias and hallways. Colleagues of second victims’ reactions included surprise and pursuit to avoid involvement.	16 Second victim
Joesten et al. (2015), USA [45]	To establish a baseline of perceived availability of institutional support services or interventions and experiences following an adverse patient safety event (PSE)	Quantitative study, The Medically Induced Trauma Support Services Staff Support Survey (MITSS) Nurses (*n* = 82), physicians (*n* = 12)	Overall, 10–30% of respondents reported that various support services or interventions were actively offered. Respondents reported having experienced several distressing symptoms after PSE, such as worrying memories (56%) and concerns about lawsuits (37%). Most of them experienced more support from colleagues than from their manager or department chair. Less than 32% felt that they could report concerns without fear of punitive action or retribution.	14 Second victim
Lewis et al. (2013), USA [46]	To report the effect of medical errors on nurses	Integrative literature review 21 articles included Inclusion criteria and search strategy described Whittemore and Knafl’s methodology used	Characteristics of units were important in nurses’ experience of medical errors. Nurse characteristics were essential, for example, number of nursing practice years. Veteran nurses were more likely to make constructive changes. Two interventions were: (1) disclosure of a medical error to the patient; and (2) support available to the nurse. Responses to the intervention outcomes were: (1) burnout, including emotional exhaustion, depersonalisation and low personal accomplishment; (2) moral distress; (3) intention to leave the profession; and (4) positive constructive changes after medical errors.	15.5 Second victim
Davies et al. (2015), UK [47]	To explore student midwives’ perceptions of what was traumatic for them and how they were supported after such events	Qualitative descriptive approach, using semi-structured interviews Student midwives (*n* = 11)	Five main themes: (1) *Students’ anxiety about entering the profession* including students being forced to adopt practices that devaluate their commitment; (2) *Existential space between a patient and qualified midwife* occupied by students, having traumatic tensions in the student role; (3) *Emergency events were traumatic* with students feeling unprepared and having too much responsibility; (4) *Aftermath of emergency events* concerning the impact of the event on students; and (5) *Learning to cope related to the way student coped with such incidents*, as well as other stresses in the role.	13.5 Second victim
Harrison et al. (2015), UK/ USA [48]	To investigate: (a) the professional or personal disruption experienced after making an error; (b) the emotional response and coping strategies used; (c) the relationship between emotions and coping strategy selections; (d) influential factors in clinicians’ responses; and (e) perceptions of organisational support	Cross-sectional, cross-country survey, The Health Professional Experience of Error Questionnaire (HPEEQ) tool Nurses (*n* = 145), physicians (*n* = 120)	Professional and personal disruption reported as a result of making an error. Negative feelings common, but positive feelings like alertness, determination and attentiveness also identified. Emotional response and coping strategy selection appeared to differ by professional group; nurses had stronger negative feelings after an error, but selection did not differ by perceived harm or location. Problem-focused coping strategies were favoured. Organisational support services perceived as helpful, especially peers, but there were fears over confidentiality. Factors that influence clinician recovery should be considered in the provision of comprehensive support programs.	17 Second victim
Seys et al. (2013b), USA [49]	To determine definitions of second victim, research the prevalence and the impact of adverse event on the second victim and the coping strategies used	Literature review 32 research articles and 9 non-research articles were identified	Second victims’ common reactions after adverse events can be emotional, cognitive and behavioural. The coping strategies used by second victims have an impact on their patients, colleagues and themselves. Defensive as well as constructive changes have been reported in practice after adverse events. It is critical that support networks are in place to protect the patient and involved healthcare providers when an adverse event occurs.	15 Second victim
Edrees and Wu (2017) USA [50]	To assess the extent of the second victim problem in acute care hospitals, the availability of emotional support services and the need for organisational support programs.	In-depth semi-structured interviews. Patient safety representatives (*n* = 43).	All participants reported that they are aware of second victim problems. Almost all agreed that hospitals should have a support program for second victims.	15,5 Second victim
Delacroix (2017), USA [51]	To discern nurse practitioners’ behaviours, perceptions and coping mechanisms in response to having made a medical error	Qualitative study, face-to-face semi-structured interviews (*n* = 10).	Four themes emerged from interviews: (1) *The paradox of error victimisation*, two subthemes were presented (fear for the patients’ welfare and fearing an uncertain professional future; (2) *The primacy of responsibility and mindfulness*, three subthemes were presented (I am responsible, acute reactions and mindfulness); (3) *Yearning for forgiveness and supportive other*, this theme was categorised in two subthemes (non-supportive just culture and seeking forgiveness and support); and (4) *Coping with a new reality is context dependent*, what was split up to two subthemes (atypical coping and constructive coping).	15.5 Second victim
Van Gerven et al. (2016), Belgium [52]	To evaluate the prevalence and content of organisations’ support systems for healthcare professionals involved in an adverse event.	Quantitative descriptive design Dutch-speaking hospitals (*n* = 59)	Thirty organisations had a systematic plan to support second victims. The chief nursing officer was seen as one of the main contact people when something went wrong. In terms of the quality of the protocols, only a minority followed part of the international resources.	16 Second and third victim

**Table 2:** “Action after adverse events” regarding first, second and third victim elements.

**FIRST VICTIM ELEMENTS**	ATTENTION OF REVEALING AN ADVERSE EVENT	HCPs listening to patients’ and family members’ concerns about an error Patients or family members fearing to speak up HCPs fearing to speak up HCPs’ empowering or disempowering patients and family members
	COMMUNICATION AFTER AN ADVERSE EVENT	Considering cultural differences in communication Providing open communication Documenting in the patient records Observing different kind of family dynamics
	FIRST VICTIM SUPPORT	Emotionally supporting patients/families after adverse events Compensation support
	COMPLETE APOLOGY FOR FIRST VICTIMS	Apology with empathy Apology being an interactive process Presenter of apology HCPs/HCOs being sorry for adverse event experience Patient forgiving an adverse event Apology including learning from an event and a change in action First victims’ trust in healthcare services
**SECOND VICTIM ELEMENTS**	SECOND VICTIM SUPPORT TYPE	Informal second victim support Formal second victim support Emotional second victim support
	SECOND VICTIMS’ COPING STRATEGIES	Individuality of second victim coping strategies Seeking second victim emotional support coping strategies Problem-solving second victim coping strategies
	SECOND VICTIM SUPPORT SERVICES	Availability of second victim support services Second victim legal and counselling support Time away second victim support Open disclosure support
	SECOND VICTIMS’ PROFESSIONAL CHANGES AFTER ADVERSE EVENTS	Defensive changes after adverse events Constructive changes after adverse events
	SECOND VICTIMS’ LEARNING ABOUT ADVERSE EVENT PHENOMENON	Second victim learning from an adverse event Learning about second victim phenomenon Learning to communicate about adverse events
**THIRD VICTIM ELEMENTS**	ORGANISATIONAL “ACTION AFTER ADVERSE EVENT” STRATEGY	Action after adverse event plan High moral communication strategy Active providing of support services Organisational apology policy Organisational learning from adverse event
	ORGANISATIONAL “ACTION AFTER ADVERSE EVENT” INFRASTRUCTURE	Action after adverse event personnel Support infrastructure Processes of “action after adverse event”
	OPEN DISCLOSURE ABOUT ADVERSE EVENT	Process of open communication Content of open disclosure
	“ACTION AFTER ADVERSE EVENT” TRAINING	Patient safety training Adverse events related training Communication after adverse events training
